# Pyridine-mediated B–B bond cleavage of tetrahydroxydiboron to synthesize n-doped SWCNTs with long-term air stability

**DOI:** 10.1038/s41598-023-48847-2

**Published:** 2023-12-11

**Authors:** Naoki Tanaka, Aoi Hamasuna, Itsuki Yamaguchi, Koichiro Kato, Tsuyohiko Fujigaya

**Affiliations:** 1 Department of Applied Chemistry, Graduate School of Engineering, Kyushu University, 744 Motooka, Nishi-ku, Fukuoka, 819-0395 Japan; 2 International Institute for Carbon Neutral Energy Research (WPI-I2CNER), Kyushu University, 744 Motooka, Nishi-ku, Fukuoka, 819-0395 Japan; 3 Center for Molecular Systems (CMS), Kyushu University, 744 Motooka, Nishi-ku, Fukuoka, 819-0395 Japan

**Keywords:** Electronic devices, Carbon nanotubes and fullerenes, Thermoelectrics

## Abstract

Neutral radicals, including carbon radicals, are highly useful chemical species for the functionalization of semiconducting materials to change their electrical and optical properties owing to their high reactivity. However, boron radicals have been limited to synthetic and reaction chemistry, with rare utilization in materials science. In this study, a mixture of tetrahydroxydiboron (B_2_(OH)_4_) and pyridine derivatives was found to act as an electron dopant for single-walled carbon nanotubes (SWCNTs) because of the electron transfer from pyridine-mediated boron radicals generated by B–B bond dissociation to neutral radicals. In particular, the radical formed from a mixture of B_2_(OH)_4_ and 4-phenylpyridine ((4-Phpy)B(OH)_2_^·^) efficiently doped electrons into the SWCNT films; thus, n-type SWCNTs with long-term air stability for more than 50 days at room temperature were prepared. Furthermore, the experimental and theoretical surface analyses revealed that the formation of stable cations from ((4-Phpy)B(OH)_2_^·^) and the efficient interaction with SWCNTs due to their high planarity served as the mechanism for their stable doping.

## Introduction

Organic neutral radicals are well-known as reactive chemical species because their singly occupied molecular orbital (SOMO) energy level is high. This leads to addition reactions or electron transfer to various semiconducting materials, including organic and inorganic semiconductors^1–7^, carbon materials^8–13^, and transition metal dichalcogenides^14–17^ In particular, carbon radicals such as alkyl and aryl radicals can be used as chemical modifiers and electron dopants for semiconducting materials to change their physical, optical, and electrical properties^5,11–14,18–21^. The generation of carbon radicals requires homolytic bond cleavage, such as the elimination of nitrogen from a diazonium ion^22^, or a hydrogen atom from a tertiary carbon atom^23^. Synthetic strategies for carbon radicals have been established to precisely control their reactivities, which is essential for applications in magnetics, electronics, optoelectronics, and spintronics^24^.

**Figure 1:**
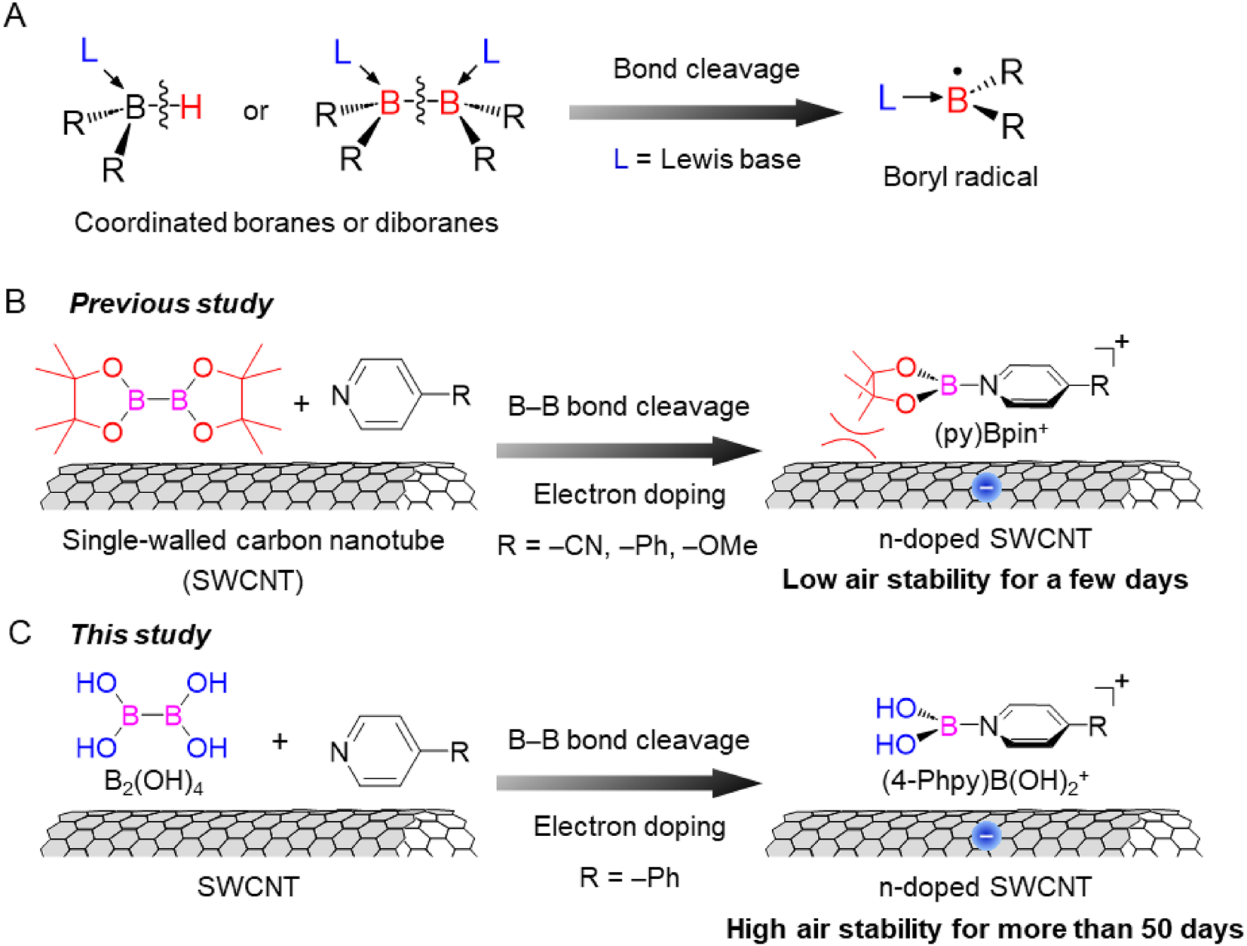
(**A**) Formation of ligated boryl radicals by B–H or B–B bond cleavages of borane or diborane compounds using Lewis bases. (**B**) Formation of n-doped SWCNTs using B_2_pin_2_ and pyridine derivatives (previous study)^30^. (**C**) Formation of highly air-stable n-doped SWCNTs using B_2_(OH)_4_ and 4-Phpy for more than 50 days (this study).

Although various carbon radicals have been used in materials chemistry, boron radicals are still primarily limited to synthesis and reaction chemistry. Neutral boron radicals have two possible electric states: a boron radical with five valence electrons and a ligated boron radical with seven valence electrons. The former is an extremely electron-deficient species that has never been isolated. In contrast, the latter was isolated by the coordination of borane or diborane compounds with Lewis bases and the subsequent cleavage of B–H or B–B bonds (Fig. 1A)^25^. For example, the coordinated boranes formed by a strong Lewis base, such as N-heterocyclic carbene and pyridine derivatives (py), are good precursors for the boron radicals because of their low B–H bonding energy^26^. These radicals react with various substrates, including alkene, alkyne, and carbonyl compounds, via radical addition reactions^27–29^. Thus, boron radicals are frequently used as boron reagents in organic synthesis to introduce boron groups into organic molecules.

As a new application for boron radicals, we have recently demonstrated the use of neutral boron radicals as electron dopants for single-walled carbon nanotubes (SWCNTs) by transferring electrons from the SOMO to the conduction band of SWCNTs, thus forming n-type SWCNTs (Fig. 1B)^30^. In this system, the reaction between bis(pinacolato)diboron (B_2_pin_2_) and 4-cyanopyridine (4-CNpy) generated a pyridine-ligated boryl radical, (4-CNpy)Bpin^**·**^, by the homolytic cleavage of the B–B bond in B_2_pin_2_ via the coordination of 4-CNpy to boron atoms^29^. Moreover, the experimental and theoretical analyses demonstrated that the electron transfer from (4-CNpy)Bpin^**·**^ to SWCNTs formed a cation, (4-CNpy)Bpin^+^, on the SWCNT surface. Notably, doping also proceeded with other pyridines, such as 4-phenylpyridine (4-Phpy) and 4-methoxypyridine (4-OMepy), which were reported to not form the corresponding radicals via B–B bond cleavage. These results indicate that the reaction of B_2_pin_2_ with Lewis bases on the SWCNT surface promotes the cleavage of its B–B bonds. However, after a few days, the obtained doped SWCNT films changed from n-type to p-type, indicating that the n-type SWCNTs were not sufficiently stable in air.

Several systems have been reported to stabilize n-type SWCNTs under atmospheric conditions for over a month^19,31,32^. These results demonstrated that the formation of stable dopant cations after doping is one of the critical factors for stabilizing n-type SWCNTs and that the dopant cations sufficiently cover the surface of the SWCNTs to prevent de-doping by oxygen. Therefore, we expected that the doping system of B_2_pin_2_ and pyridine derivatives would not provide sufficient dopant cations to cover the surface of the SWCNTs because of the steric repulsion between the four methyl groups of the Bpin unit and SWCNTs.

In this study, we employed tetrahydroxydiboron (B_2_(OH)_4_) having smaller molecular size than B_2_pin_2_ to generate boron radicals through pyridine ligation in order to improve the coverage efficiency of SWCNT doping. Consequently, we achieved long-term air stability of the n-type SWCNTs at room temperature by doping with B_2_(OH)_4_ and 4-Phpy (Fig. 1C). Based on the experimental and theoretical analyses of the doped SWCNT film, we found that the boronic acid group and phenyl substituent of the dopant cation contributed to the air stability of the n-doped SWCNTs.

## Results and discussion

### Electron doping of SWCNT using boryl radicals

**Figure 2:**
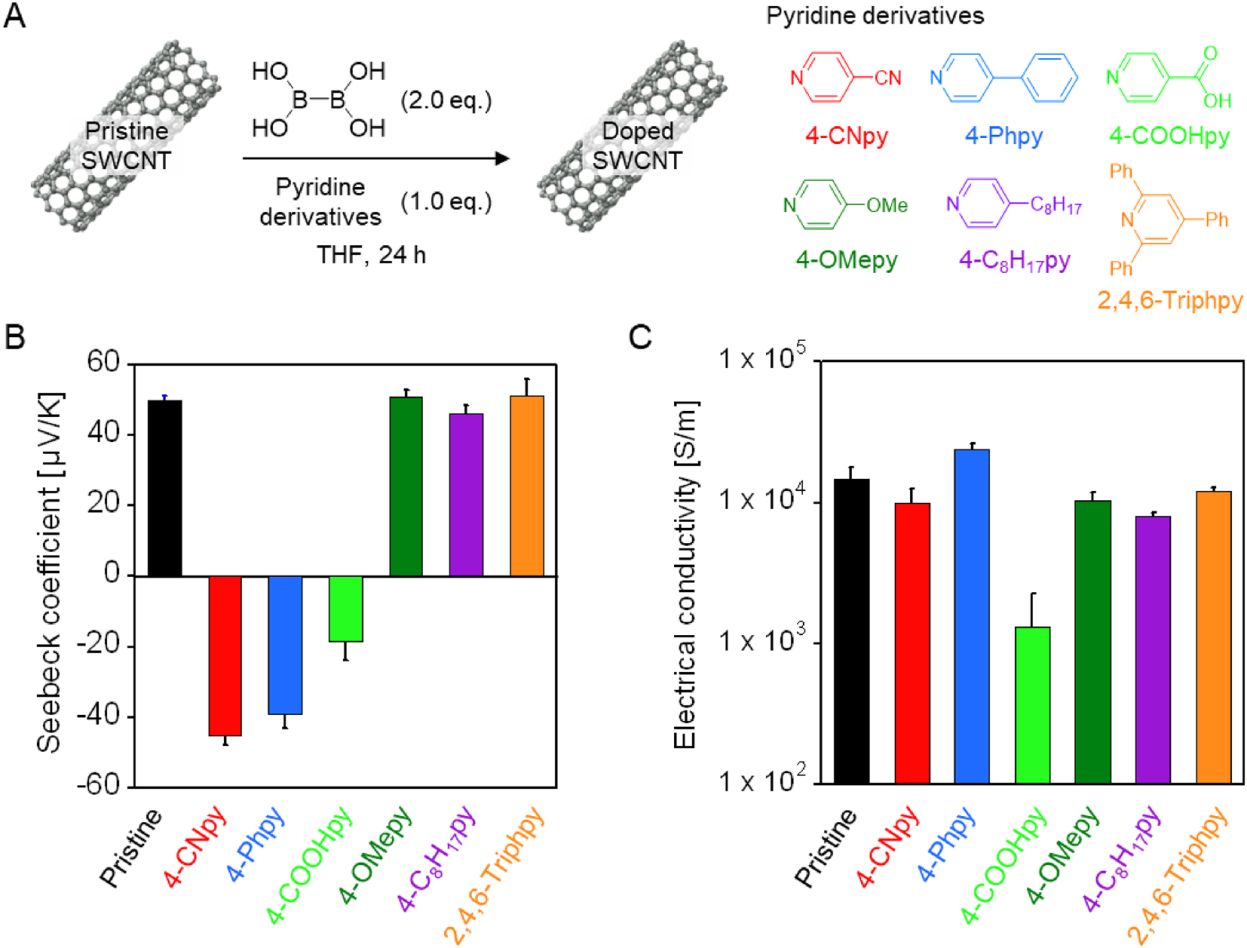
(**A**) Electron doping of SWCNTs using a mixture of B_2_(OH)_4_ and pyridine derivatives. (**B**) Seebeck coefficient and (**C**) electrical conductivity of pristine SWCNT films and SWCNT films doped by B_2_(OH)_4_ and pyridine derivatives at 30 °C. Error bars represent the standard deviation of technical replicates.

The SWCNT films were fabricated by filtrating the SWCNT dispersion in *N*-methylpyrrolidone (NMP), and the pristine SWCNT films (15 ± 5 µm in thickness) were immersed in a tetrahydrofuran (THF) solution of B_2_(OH)_4_ (4.0 mM) and pyridine derivatives (2.0 mM) under nitrogen and then shaken for 24 h at 30 °C (Fig. 2A). After drying the SWCNT films, the Seebeck coefficient and electrical conductivity of the doped SWCNT films were evaluated. In this study, pyridine derivatives 4-CNpy, 4-Phpy, 4-OMepy, 4-pyridinecarboxylic acid (4-COOHpy), 4-octylpyridine (4-C_8_H_17_py), and 2,4,6-triphenylpyridine (2,4,6-Triphpy) were investigated. As shown in Fig. 2B, the pristine SWCNT films exhibit a positive Seebeck coefficient of 50.0 μV/K at 30 °C, indicating their p-type nature owing to hole doping with oxygen in air. In contrast, doping by the mixture of B_2_(OH)_4_ with 4-CNpy, 4-Phpy, and 4-COOHpy yields a negative Seebeck coefficient of − 45.2 μV/K, − 38.9 μV/K, and − 18.6 μV/K, respectively (Fig. 2B). SWCNT films solely doped with B_2_(OH)_4_ or these pyridines exhibit a positive Seebeck coefficient (Fig. S1), indicating the importance of combining B_2_(OH)_4_ and pyridine for electron doping. We considered that the reaction of pyridines with B_2_OH_4_ produced pyridine-ligated boryl radicals ((py)B(OH)_2_^**·**^) by the B–B bond cleavage on the surface of SWCNT, resulting in the n-doped SWCNT films based on the electron transfer from the radicals to SWCNTs. For these combinations, an electron-withdrawing group (EWG) and an aromatic substituent at the *para*-position of pyridine might stabilize (py)B(OH)_2_^**·**^ owing to the delocalization of the radical (captodative effect)^33^, and effective electron transfer from the radical to the SWCNTs occurred. From the Raman spectroscopy of these films, the intensity of the D band at 1331 cm^−1^ in reference to that of the G band at 1580 cm^−1^ (G/D ratio) was unchanged after doping (Fig. S2), clearly indicating the doping reaction did not involve the covalent bond formation. In contrast, Seebeck coefficients and electrical conductivities of the SWCNT films doped with 4-OMepy, 4-C_8_H_17_py, and 2,4,6-Triphpy with B_2_(OH)_4_ were comparable to those of pristine SWCNT films (Fig. 2B). The results indicate that any doping reaction did not occur. For these pyridine derivatives, the formation of the boryl radical might be unfavorable due to the weak captodative effect caused by electron-donating substitutional groups for 4-OMepy and 4-C_8_H_17_py and steric hindrance for 2,4,6-Triphpy.

In terms of electrical conductivity, the SWCNT film doped with B_2_(OH)_4_ and 4-Phpy exhibited the highest value of 2.4 × 10^4^ S/m, indicating the most efficient electron injection from the generated radicals (Fig. 2C). The doping ability of the radicals is related to their SOMO energy. In fact, the calculated SOMO energies of (4-CNpy)B(OH)_2_^**·**^, (4-Phpy)B(OH)_2_^**·**^, and (4-COOHpy)B(OH)_2_^**·**^ in vacuum at UB3LYP/6–31++G(d,p) are − 4.38 eV, − 3.59 eV, and − 4.42 eV, respectively (Fig. S3), in good agreement with the trend for electrical conductivity. We also studied the effect of the doping concentration using B_2_OH_4_ and 4-Phpy and found that increase of the concentrations increased the conductivity of the doped SWCNT films, while the absolute Seebeck coefficient decreased (Fig. S4), suggesting the controllability of the dope levels by dopant concentrations. Noted that these concentrations were much lower than that of the previously reported system using B_2_pin_2_ (26.3 mM)^30^. The doping of SWCNT by B_2_pin_2_/4-Phpy under the same conditions (B_2_pin_2_:4-Phpy = 2.0 mM:1.0 mM) did not provide n-doped SWCNT films (Fig. S5), suggesting that the B-B bond cleavage of B_2_pin_2_ did not undergo efficiently due to its higher B–B binding energy than that of B_2_OH_4_^34^.

### Surface analysis of n-doped SWCNTs using boryl radicals

**Figure 3:**
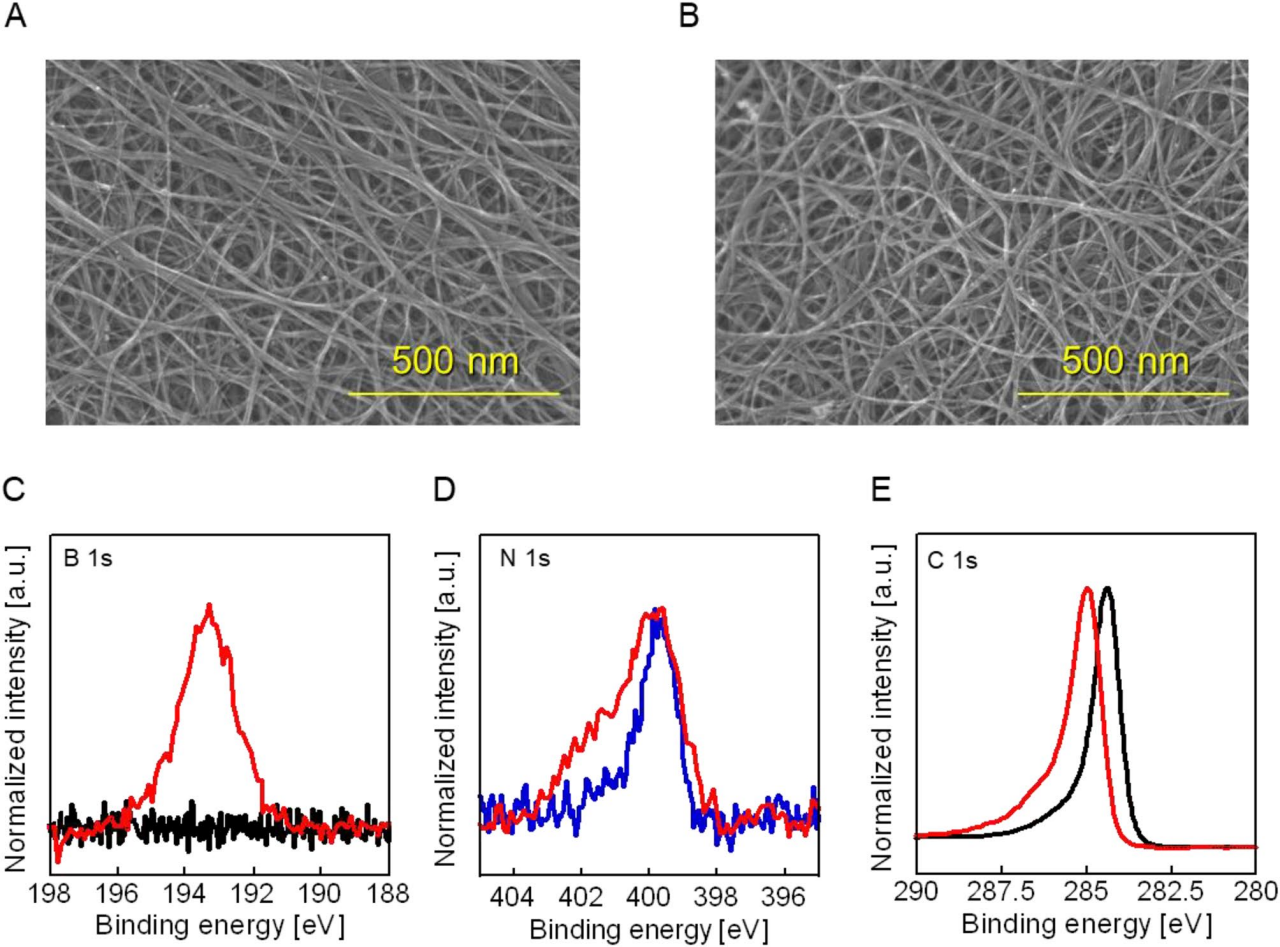
SEM images of (**A**) pristine SWCNT films and (**B**) doped SWCNT films by B_2_OH_4_ (4.0 mM)/4-Phpy (2.0 mM). X-ray photoelectron spectroscopy (XPS) narrow scans of (**C**) B 1 s, (**D**) N 1 s, and (**E**) C 1 s of the n-doped SWCNT films using B_2_OH_4_/4-Phpy (red). Black and blue lines represent the pristine SWCNT films and SWCNT films doped with 4-Phpy.

To characterize the film structure, scanning electron microscopy (SEM) of B_2_OH_4_/4-Phpy-doped SWCNT films with different concentrations (Fig. 3A,B, Fig. S6) was performed. All films formed similar bundle structures with network morphology, indicating that the dopant layer was sufficiently thin and the doping process did not change the film structure^35^. X-ray photoelectron spectroscopy (XPS) was performed on the SWCNT films doped with B_2_OH_4_ and 4-Phpy (B_2_OH_4_/4-Phpy) to characterize the chemical structure of the dopant on the doped SWCNT film (Fig. 3). The presence of the B 1 s peak at 193 eV (Fig. 3C) and the N 1 s peak at approximately 400 eV (Fig. 3D) indicates the presence of the dopant compound (Fig. S7). A narrow scan of the N 1 s region reveals a broad peak composed of two peaks at 400 eV and 402 eV, whereas the control SWCNT film doped with only 4-Phpy shows a sharper peak centered at 400 eV, attributed to the C–N bond (Fig. 3D). Because the peak at 402 eV is attributed to quaternary ammonium^36,37^, the result indicates the formation of dopant cations ((4-Phpy)B(OH)_2_^+^) on the SWCNT surface because of electron transfer from 4-Phpy-B(OH)_2_^**·**^ to the SWCNTs. In addition, the peaks of the doped SWCNT films shift to a higher binding energy by 0.4 eV in the C 1 s region after doping (Fig. 3E), indicating that the SWCNTs receive electrons and, consequently, the Fermi level increases. Notably, the control SWCNT film doped solely with B_2_OH_4_ does not exhibit any peaks in the B 1s region (Fig. S8), whereas the SWCNT film doped with B_2_OH_4_/4-Phpy exhibits a strong peak at 193 eV. This indicates that B_2_OH_4_ is removed by sublimation under ultrahigh-vacuum conditions, whereas the dopant cations are stably adsorbed on the SWCNT surface (Fig. 3C).

To elucidate the dopant structure on the SWCNT surface after doping, the B_2_OH_4_/4-Phpy-doped films were washed with deuterium THF and the ^1^H NMR of the solutions were measured. We observed the peaks assignable to B_2_OH_4_ and 4-Phpy in aromatic regions, whereas the signals from (4-Phpy)B(OH)_2_^+^ were not observed (Fig. S9). The result indicates the strong interaction between dopant cation and negatively-charged n-type SWCNT surface.

### Air stability of n-doped SWCNT films

**Figure 4:**
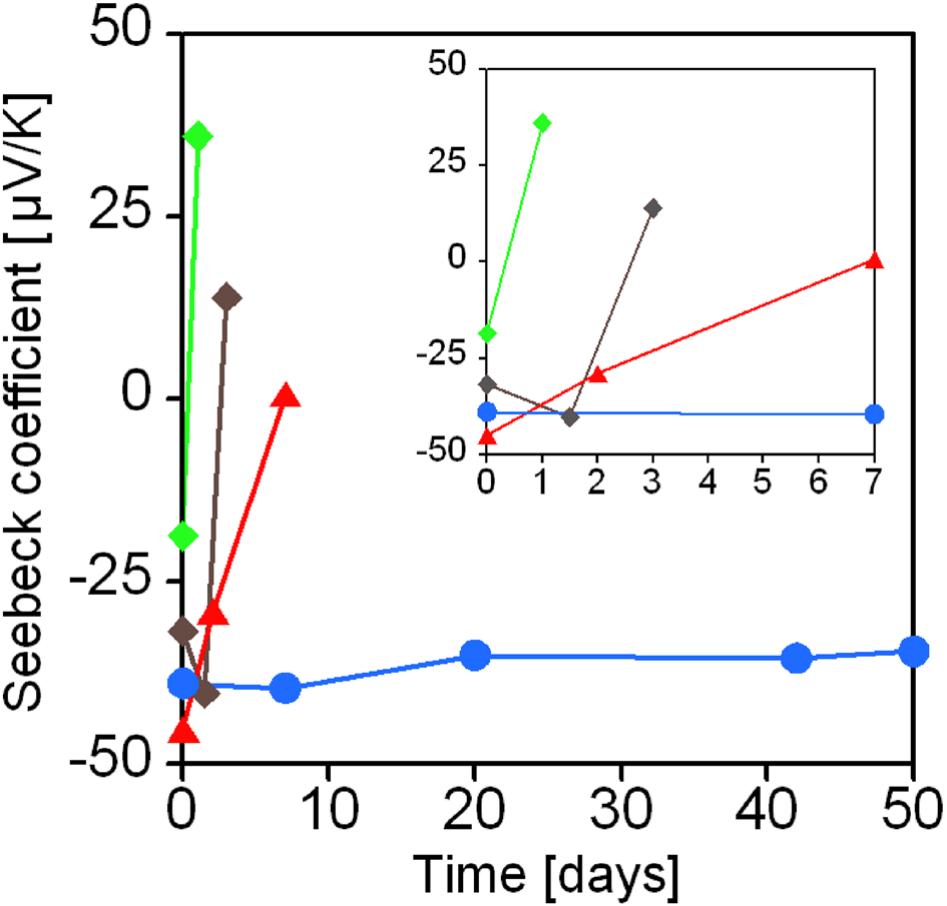
Time course of the Seebeck coefficient of the SWCNT film doped with B_2_(OH)_4_/4-CNpy (red), B_2_(OH)_4_/4-COOHpy (green), and B_2_(OH)_4_/4-Phpy (blue). Time course of the Seebeck coefficient of the SWCNT film doped with B_2_pin_2_/4-Phpy (gray) was plotted for comparison^30^.

The time course of the Seebeck coefficient was monitored to determine the stability of the doped SWCNT films (Fig. 4). The SWCNT films doped with B_2_OH_4_/4-Phpy exhibited long-term air stability for more than 50 days, whereas the SWCNT films doped with B_2_OH_4_/4-CNpy and B_2_OH_4_/4-COOHpy reverted to the p-type within a few days. A primary requirement of stability is the formation of stable dopant cations on the SWCNT surface. In this system, the dopants possessed a delocalized positive charge on the pyridine moiety after the doping (Fig. S10), thus an electric repulsion occurs when the cationic pyridine possesses EWG that have cationic (δ^+^) carbons. We consider that such a repulsion lowered the stability of the dopant cations for B_2_OH_4_/4-CNpy and B_2_OH_4_/4-COOHpy. On the other hand, the phenyl group stabilized the cationic pyridine probably because of the π-electron donation from the phenyl group to cationic pyridine. In fact, the molecular orbital of HOMO-7 calculated by density functional theory (DFT) demonstrates π donation from the phenyl group to the cationic pyridine (Fig. S11).

### Theoretical calculation of n-doped SWCNT using boryl radicals

Notably, although (4-Phpy)B(OH)_2_^**·**^ and (4-Phpy)Bpin^**·**^ exhibit similar SOMO energies, the SWCNT films doped with B_2_pin_2_/4-Phpy return to the p-type nature in one week (Fig. 4, Fig. S3).^30^ This indicates that the boronic acid group of the dopant cation contributes to the enhancement of the air stability of the n-doped SWCNT film.

**Figure 5:**
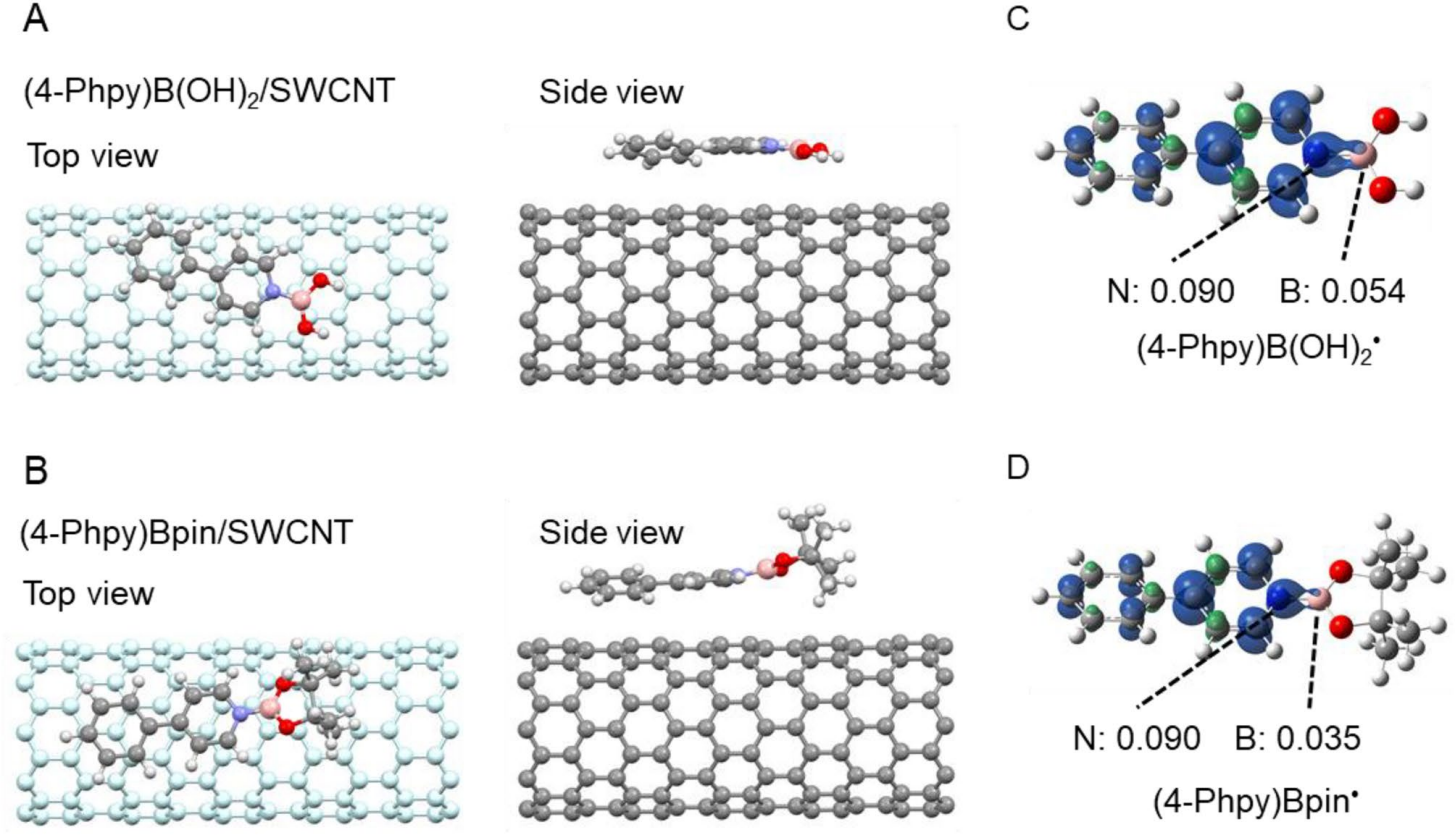
Optimized geometries of (**A**) (4-Phpy)B(OH)_2_/SWCNT (10,0) and (**B**) (4-Phpy)Bpin/SWCNT (10,0). Spin density of the structures of (**C**) (4-Phpy)B(OH)_2_^**·**^ and (**D**) (4-Phpy)Bpin^**·**^. Boron, carbon, nitrogen and oxygen atoms are colored pink, gray, blue and red, respectively.

To understand the stability effect, we performed DFT calculation on the SWCNT (10,0) complexes with (4-Phpy)B(OH)_2_^**·**^ and (4-Phpy)Bpin_2_^**·**^ using gaussian software. Figure 5A,B show top and side views of the optimized structures of the SWCNT complexes. The distance between the substituted phenyl group and the SWCNT is 3.29 Å for (4-Phpy)B(OH)_2_/SWCNT and 3.24 Å for (4-Phpy)Bpin/SWCNT, respectively, reflecting π-stacking between the phenyl group and SWCNT. In the skeleton around the boron, (4-Phpy)B(OH)_2_ forms a planar structure on the SWCNT surface, while (4-Phpy)Bpin is found to be warped with respect to the SWCNT surface due to the steric hindrance of Bpin. The energy gains after doping were not significantly different for each complex (Table S1). However, the charge of the dopants after doping exhibits 0.21e for (4-Phpy)B(OH)_2_ and 0.10e for (4-Phpy)Bpin, respectively, indicating that (4-Phpy)B(OH)_2_^**·**^ can effectively transfer electrons to SWCNT (Table S2). Together with the effective stacking of between (4-Phpy)B(OH)_2_ and SWCNTs due to the structural planarity of (4-Phpy)B(OH)_2_, slightly larger spin density of boron of (4-Phpy)B(OH)_2_^**·**^ (0.054, Fig. 5C) than that of (4-Phpy)Bpin^**·**^ (0.035, Fig. 5D) might facilitate the superior electron transfer. Although direct observation of the boryl radicals by such as electron spin resonance (ESR) has not succeeded yet, these calculation offers significant insights to elucidate the doping reaction as well as the stability mechanism.

To further evaluate the amount of dopant on the doped SWCNT surface, thermogravimetric analysis (TGA) of the B_2_OH_4_/4-Phpy-doped and B_2_pin_2_/4-Phpy-doped SWCNT films were performed. The weight loss of B_2_OH_4_/4-Phpy-doped films (30wt.%) and B_2_pin_2_/4-Phpy-doped films (15wt.%) from 200 to 500 °C correspond to the weight loss of (4-Phpy)B(OH)_2_^+^ and (4-Phpy)Bpin^+^, respectively, those includes the unreacted diborane and pyridine (Fig. S12), while the weight losses at 30–200 °C were originated from the hydrated water. Larger weight losses for B_2_OH_4_/4-Phpy-doped films (30 wt%) than B_2_pin_2_/4-Phpy-doped films supports higher coverage of the B_2_OH_4_/4-Phpy-doped films by dopant cations. We believe the present results will contribute for the development of unique materials utilizing boron radicals.

## Conclusion

A new electron doping method for SWCNTs has been developed using B_2_OH_4_ and pyridine derivatives based on homolytic B–B bond cleavage. Using B_2_OH_4_ and 4-Phpy, the doping proceeds effectively because of the high SOMO level of generated (4-Phpy)B(OH)_2_^**·**^, providing long-term air-stable n-doped SWCNT films for over 50 days. Moreover, surface analysis of the n-doped SWCNT film revealed that (4-Phpy)B(OH)_2_ cations were formed as counter cations of the n-doped SWCNT, which were stable cations owing to the π-electron donation to a cationic pyridine moiety. The theoretical calculation found that (4-Phpy)B(OH)_2_^**·**^ can proceed effectively electron doping to SWCNT owing to the high spin density of boron and the small steric hindrance of the BOH units. These results provide a novel dopant design for future SWCNT-based electronic devices.

## Methods

### Materials

SWCNTs (Meijo-eDIPS) with a diameter of 1.5 ± 0.5 nm were purchased from Meijo Nano Carbon. THF, NMP, 4-pyridinecarboxylic acid, and 4-octylpyridine were purchased from FUJIFILM Wako Pure Chemical Corp. (Tokyo, Japan). Bis(pinacolato)diboron, tetrahydroxydiboron, 4-cyanopyridine, 4-phenylpyridine, 4-methoxypyridine, and 2,4,6-triphenylpyridine were purchased from Tokyo Chemical Industry (Tokyo, Japan).

### Characterization

XPS (AXIS Ultra, Shimadzu, Kyoto, Japan) was performed at room temperature, with indium as the substrate. An Au film (Au 4f7/2, 84.140 eV) was measured using each sample as an internal standard. Scanning electron microscopy (SEM; SU-9000, Hitachi High Technologies, Tokyo, Japan) was performed at an accelerating voltage of 15 kV. ^1^H NMR spectra were recorded using a JEOL JNM-ECZ400 (400 MHz). Raman spectra were recorded at an excitation wavelength of 533 nm using a Raman touch spectrometer (Nanophoton Corporation, Osaka, Japan). The in-plane electrical conductivity and in-plane Seebeck coefficient were measured using a Seebeck coefficient/electric resistance measurement system (ZEM-3, ADVANCE RIKO, Yokohama, Japan) in a helium atmosphere at ~ 0.01 MPa from 30 to 100 °C. Thermogravimetric analysis (TGA; TG/DTA7300, Hitachi High-Technologies, Tokyo, Japan) was performed using a ceramic pan at 30–900 °C (10 °C/min), under nitrogen gas flow (300 mL/min).

### Theoretical calculation

Density functional theory (DFT) calculations were performed using Gaussian 16 program package^38^. Geometry optimizations of boryl radicals were performed using the UB3LYP functional with a basis set of 6-31G++(d,p) for all atoms. Geometry optimization of (4-Phpy)B(OH)_2_^+^ was calculated by the B3LYP functional with a basis set of 6-31G(d,p) for all atoms. (4-Phpy)B(OH)_2_/SWCNT and (4-Phpy)Bpin/SWCNT composites were performed using the UB3LYP functional with a basis set of 6-31G+(d,p) and the empirical dispersion correction by Grimme D3.

## Fabrication of SWCNT sheets

SWCNTs (5.0 mg) were dispersed in NMP (250 mL) using a bath-type sonicator (Branson 5010) for 1 h. The dispersion was filtered through a polytetrafluoroethylene membrane (pore diameter: 1.0 μm). The obtained film was removed from the membrane and washed by dipping it in methanol to remove residual NMP, followed by vacuum-drying at 80 °C for 8 h. The thickness of the films was 15 ± 5 μm. The freestanding SWCNT sheets were cut into specified sizes using scissors.

## Doping of SWCNTs with B_2_(OH)_4_ and pyridine derivatives

The pristine SWCNT film was immersed in a THF (4.0 mL) solution of B_2_(OH)_4_ (1.4 mg, 1.67 × 10^−2^ mmol) and pyridine derivatives (0.773 × 10^−2^ mmol) at 30 °C under nitrogen flow; the mixture was shaken for 24 h. After removing the films from the solution, the doped SWCNT films were vacuum-dried at room temperature for 8 h. Noted that the unreacted B_2_(OH)_4_ and pyridine derivatives were not removed to study the effect of doping concentration.

### Supplementary Information

Supplementary Information.

## Data Availability

The datasets used and/or analyzed during the current study are available from the corresponding author upon reasonable request.
