# Supplementary material for: Pyridine-mediated B–B bond cleavage of tetrahydroxydiboron to synthesize n-doped SWCNTs with long-term air stability
Source: Sci Rep. 2023 Dec 11;13:21926. doi: 10.1038/s41598-023-48847-2 (PMC10713570; doi:10.1038/s41598-023-48847-2)
Supplement: Supplementary file 1 — Supplementary Information. [file 41598_2023_48847_MOESM1_ESM.pdf]

Electronic Supplementary Information

for

Pyridine-mediated B–B bond cleavage of tetrahydroxydiboron to synthesize n-doped SWCNTs with long-term air stability

Naoki Tanaka, Aoi Hamasuna, Itsuki Yamaguchi, Koichiro Kato, and Tsuyohiko Fujigaya**

Corresponding Author

Naoki Tanaka: tanaka.naoki.468@m.kyushu-u.ac.jp

Tsuyohiko Fujigaya: fujigaya.tsuyohiko.948@m.kyushu-u.ac.jp

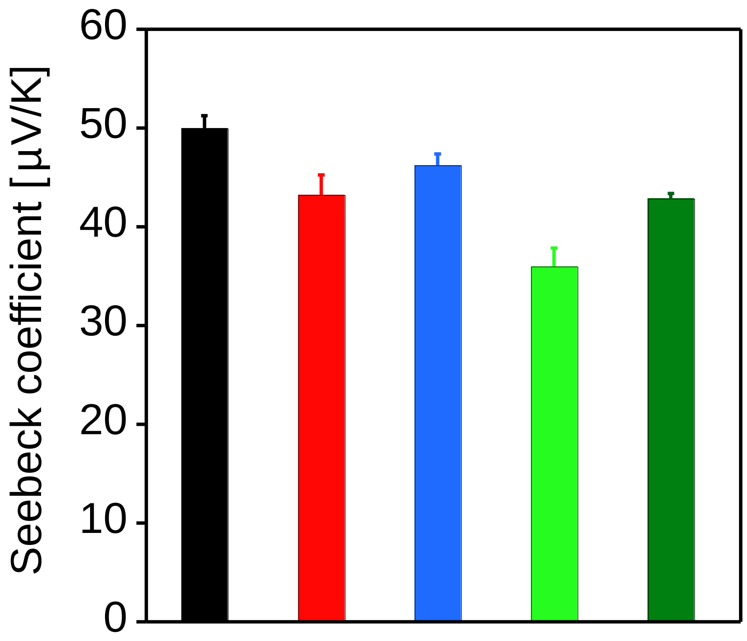

Figure S1. Seebeck coefficient of the doped SWCNT films using 4-CNpy (red), 4-Phpy (blue), 4-COOHpy (yellow-green), and $B_2(OH)_4$ (green) in air. Black shows pristine SWCNT films.

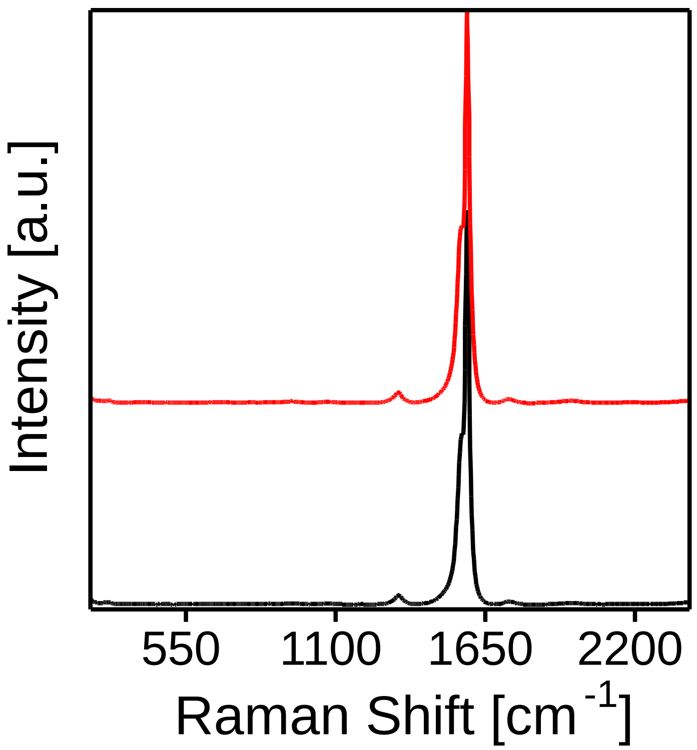

Figure S2. Raman spectra of pristine SWCNT films (black) and doped SWCNT films by B_2OH_4 (4.0 mM) and 4-Phpy (2.0 mM) (red).

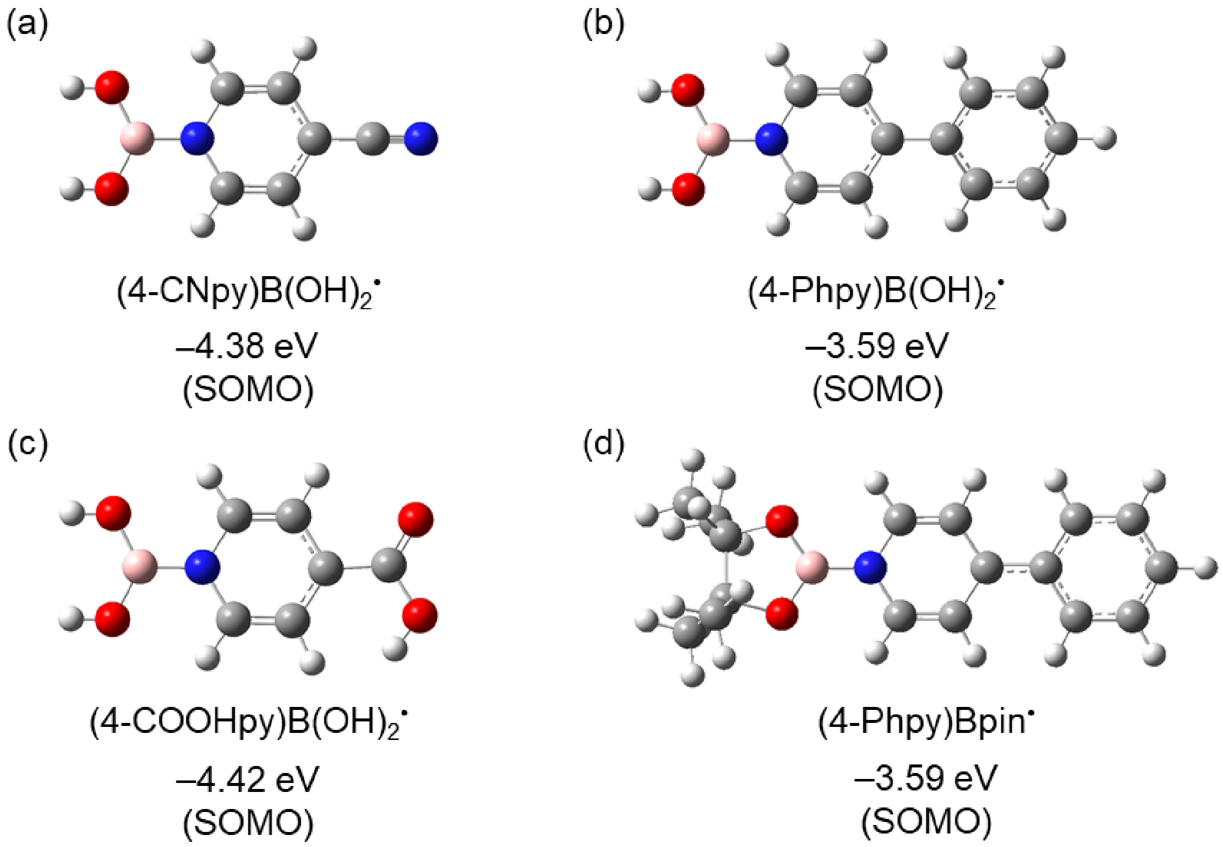

Figure S3. Energy levels of (4-CNpy)B(OH)$_2^{\bullet}$, (4-Phpy)B(OH)$_2^{\bullet}$, (4-COOHpy)B(OH)$_2^{\bullet}$, and (4-Phpy)Bpin$^{\bullet}$ in optimized geometries [UB3LYP/6-31G++(d,p) level of theory].

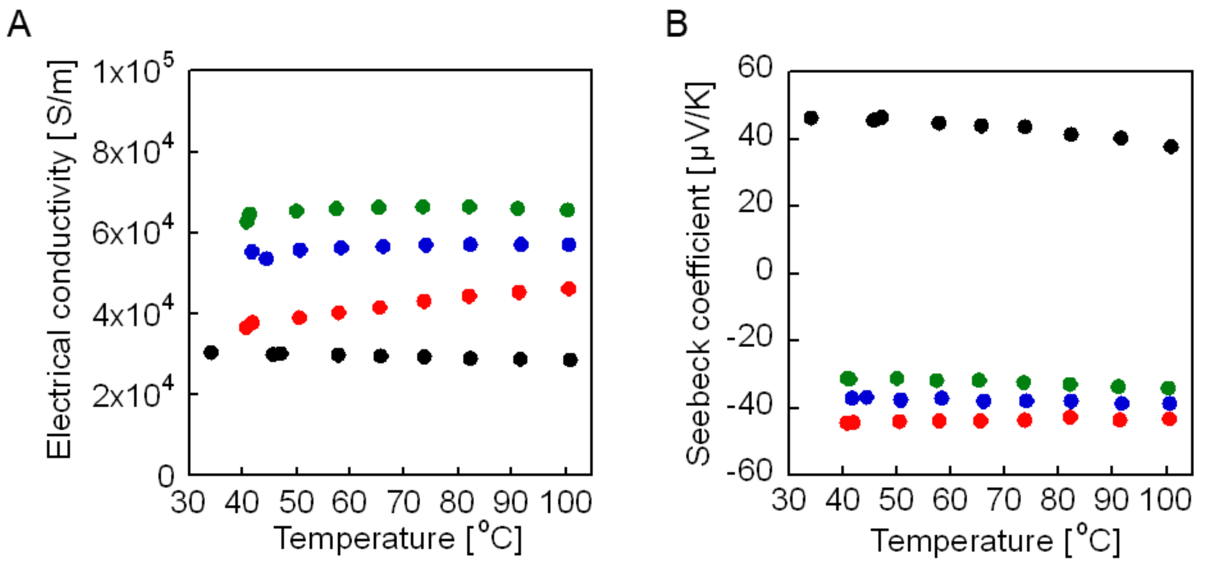

Figure S4. B_2OH_4 and 4-Phpy concentration dependence of (**A**) electrical conductivity and (**B**) Seebeck coefficient for pristine SWCNT films (black) and doped SWCNT films with B_2OH_4/4-Phpy. Doping conditions were B_2OH_4:4-Phpy = 2.0 mM:1.0 mM for red color, B_2OH_4:4-Phpy = 4.0 mM:2.0 mM for blue color, B_2OH_4:4-Phpy = 6.0 mM:3.0 mM for green color.

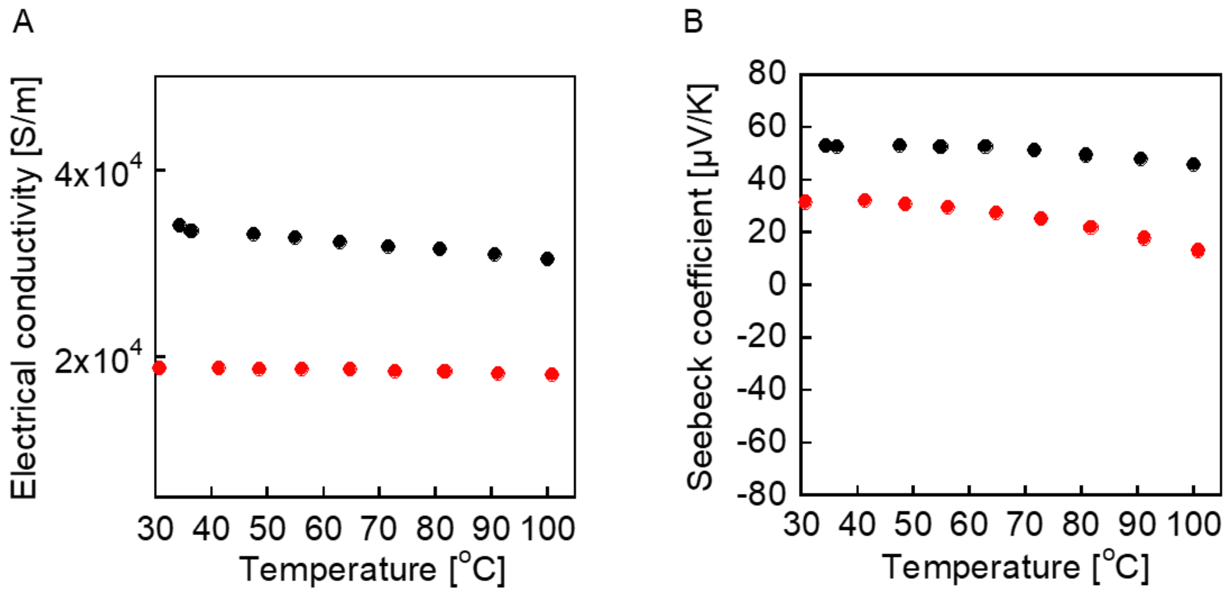

Figure S5. (**A**) electrical conductivity and (**B**) Seebeck coefficient for pristine SWCNT films (black) and doped SWCNT films (red) with B_2pin_2/4-Phpy. Doping conditions were B_2pin_2:4-Phpy = 2.0 mM:1.0 mM.

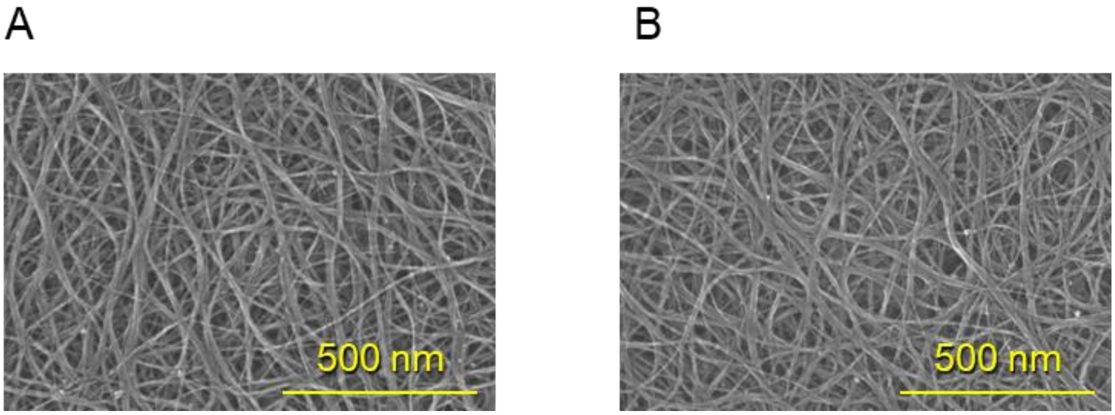

Figure S6. SEM images of doped SWCNT films with different concentrations of B_2OH_4/4-Phpy. (**A**) B_2OH_4:4-Phpy = 2.0 mM:1.0 mM, (**B**) B_2OH_4:4-Phpy = 6.0 mM:3.0 mM.

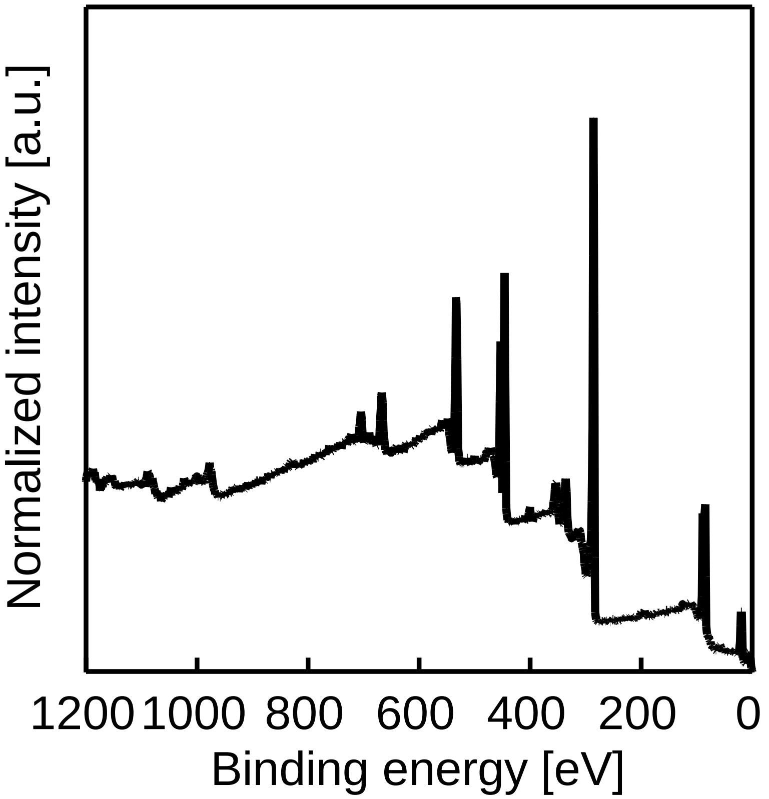

Figure S7. XPS survey scans of the n-doped SWCNT films using B_2OH_4/4-Phpy.

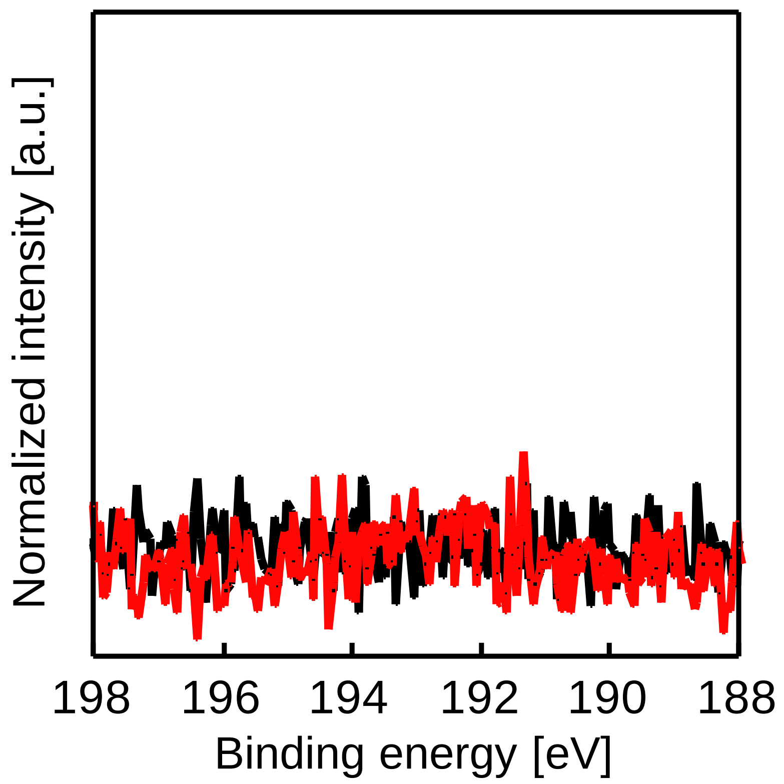

Figure S8. XPS narrow scans of B 1s for pristine SWCNT film (black) and SWCNT film after immersing in $B_2(OH)_4$ solution (red).

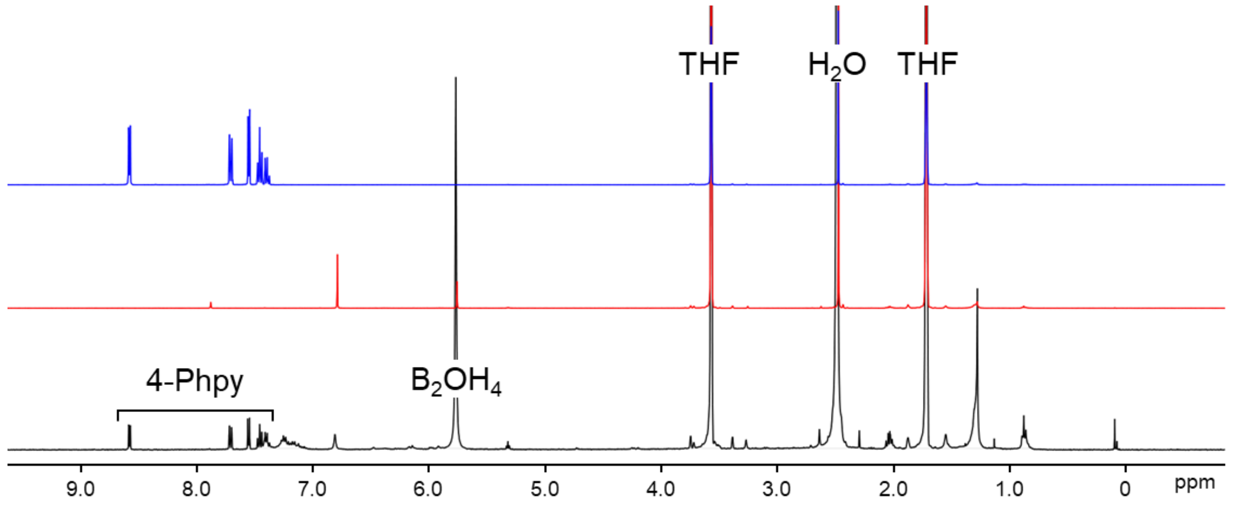

Figure S9. ^{1}H NMR spectra of extracts from doped SWCNT films with B_2OH_4/4-Phpy (black), B_2OH_4 (red), 4-Phpy (blue) in THF-d_8 solution (400 MHz, 25 °C).

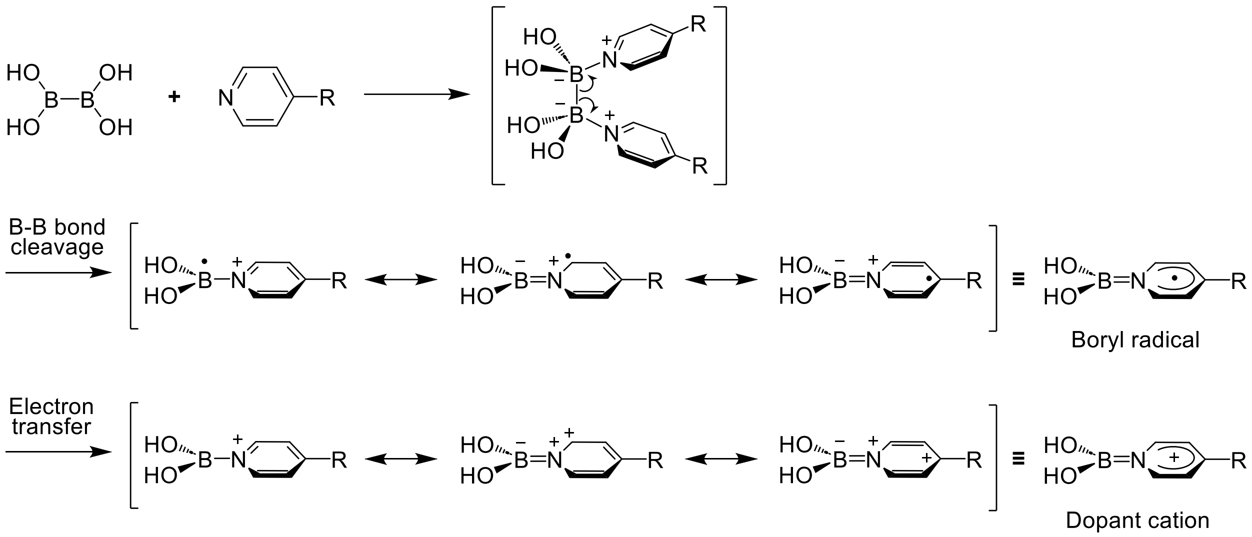

Figure S10. Resonance structures of boryl radicals and the dopant cations.

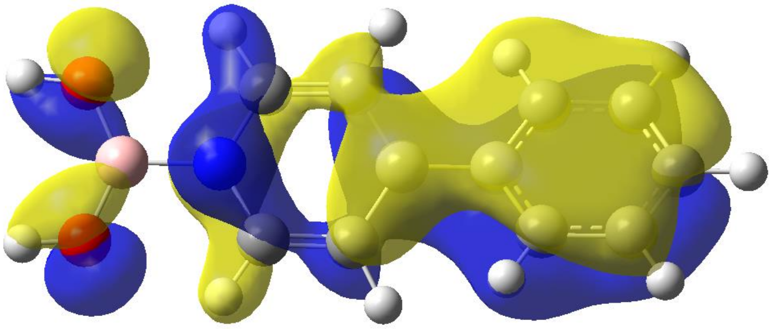

Figure S11. Molecular orbital of HOMO–7 of $(4\text{-Phpy})B(OH)_2^+$ in the optimized geometry [B3LYP/6-31G(d,p)].

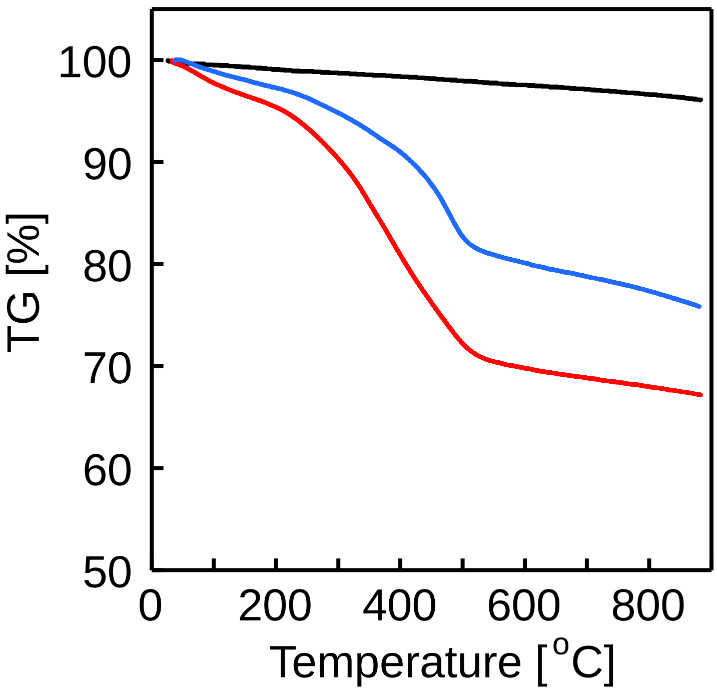

Figure S12. Thermogravimetric analysis of pristine SWCNT film (black) and n-doped SWCNT films using B_2pin_2/4-Phpy (blue) and B_2OH_4/4-Phpy (red).

Table S1. Energy gain after doping of SWCNT using B_2OH_4/4-CNpy, B_2OH_4/4Phpy, B_2pin_2/4-CNpy, and B_2pin_2/4-Phpy.

	Complex [Hartree]	SWCNT [Hartree]	Dopant [Hartree]	Energy gain [Hartree]	Energy gain [kcal/mol]
(4-CNpy)B(OH)$_2$ /SWCNT	–8151.952	–7634.718	–517.194	–0.040	–25.154
(4-Phpy)B(OH)$_2$ /SWCNT	–8290.791	–7634.718	–656.023	–0.050	–31.290
(4-CNpy)Bpin /SWCNT	–8386.671	–7634.718	–751.899	–0.053	–33.223
(4-Phpy)Bpin /SWCNT	–8525.510	–7634.718	–890.729	–0.063	–39.413

Table S2. Charge change of dopants before and after doping.

	Molecular charge in isolation [e]	Molecular charge after doping [e]
$(4\text{-CNpy})B(OH)_2^{\bullet}$	0	+0.02
$(4\text{-Phpy})B(OH)_2^{\bullet}$	0	+0.21
$(4\text{-CNpy})Bpin^{\bullet}$	0	+0.10
$(4\text{-Phpy})Bpin^{\bullet}$	0	+0.01
